# The peak of the ‘Blue Monday’ depression and winter blues.

**DOI:** 10.1192/j.eurpsy.2024.1099

**Published:** 2024-08-27

**Authors:** A. Botica, M. Baković, M. Strikić, A. Delić, T. Glavina

**Affiliations:** ^1^Department of psychiatry, University Hospital of Split, Split; ^2^Ugljan, Psychiatric Hospital, Zadar; ^3^Public Health Institute of Split and Dalmatian County, split, Croatia

## Abstract

**Introduction:**

For many people, January is the most depressing month of the year. “Blue Monday” encompasses the generally accepted belief that Monday is the hardest day of the week compared to Friday and Saturday, which are the most anticipated days of the week. The connection between the color blue and Monday is in the emotional stage, which is presented as emotional anger. The third Monday in January is currently known as the most depressing day of the year. Speaker Cliff Arnall was the first to declare that day in 2014. The theory says that this is the time of the year when respiratory diseases are common, the day is shorter, the weather conditions are worse, and the time when people are burdened with guilt about whether they will achieve their New Year’s resolutions.

**Objectives:**

The aim of this work was to investigate that on third Monday in January there were more suicide attempts and that there were more depressive disorders in emergency psychiatric admissions.

**Methods:**

In the research, we included participants who were examined at the Emergency Psychiatric Admission of the Clinical Hospital Center in Split, in the period from 2019. until 2023. Inclusion criteria were respondents of both sexes, examined in the outpatient clinic on Mondays in January for five years.

**Results:**

There were 198 of them in total. The primary outcome of the research is to determine the occurrence (incidence) of psychological deterioration in patients diagnosed with the anxiety-depressive spectrum. The secondary research outcomes are of a descriptive nature, patient follow-up, examination outcome, and psychiatric heredity.

**Conclusions:**

For now, there are no strong scientific foundations that justify the formula of “the most depressing day” of the year, some scientists believe that it is a marketing trick to achieve higher tourist revenues. However, the post-holiday period can have an impact on individuals.

**Disclosure of Interest:**

None Declared

